# Experimental and computational chemical studies on the corrosion inhibitive properties of carbonitrile compounds for carbon steel in aqueous solutions

**DOI:** 10.1038/s41598-021-00701-z

**Published:** 2021-11-04

**Authors:** Abd El-Aziz S. Fouda, Abdelmonem H. El-Askalany, Ahmed F. S. Molouk, Niveen S. Elsheikh, Ashraf S. Abousalem

**Affiliations:** 1 Chemistry Department, Faculty of Science, Mansoura University, El-Mansoura, Egypt; 2 Quality Control Laboratory, Operations Department, JOTUN, Cairo, Egypt

**Keywords:** Corrosion, Chemical physics

## Abstract

The present work aims to study 6-amino-4-aryl-2-oxo-1-phenyl-1,2-dihydropyridine-3,5-dicarbonitrile derivatives namely: 6-Amino-2-oxo-1,4-diphenyl-1,2-dihydropyridine-3,5-dicarbonitrile (PdC-H), 6-Amino-2-oxo-1-phenyl-4-(p-tolyl)-1,2-dihydropyridine-3,5-dicarbonitrile (PdC-Me) and 6-Amino-4-(4-hydroxyphenyl)-2-oxo-1-phenyl-1,2-dihydropyridine-3,5-dicarbonitrile (PdC-OH) as corrosion inhibitors to provide protection for carbon steel in a molar hydrochloric acid medium. Chemical measurements such as (weight loss) and electrochemical techniques such as (Potentiodynamic polarization, electrochemical impedance spectroscopy, and Electron frequency modulation) were applied to characterize the inhibitory properties of the synthesized derivatives. The adsorption of these derivatives on the carbon steel surface was confirmed by Attenuated Total Refraction Infrared (ATR-IR), Atomic Force Microscope (AFM), and X-ray Photoelectron Spectroscopy (XPS). Our findings revealed that the tested derivatives have corrosion inhibition power, which increased significantly from 75.7 to 91.67% on the addition of KI (PdC-OH:KI = 1:1) to inhibited test solution with PdC-OH derivative at 25 °C. The adsorption process on the metal surface follows the Langmuir adsorption model. XPS analysis showed that the inhibitor layer consists of an iron oxide/hydroxide mixture in which the inhibitor molecules are incorporated. Computational chemical theories such as DFT calculations and Mont Carlo simulation have been performed to correlate the molecular properties of the investigated inhibitors with experimental efficiency. The theoretical speculation by Dmol3 corroborates with the results from the experimental findings.

## Introduction

Corrosion is a global industrial concern worldwide for decades due to its deteriorating effect on materials not only metallic materials, but also other materials used in the construction industry. Corrosion causes significant economic loss and jeopardizes human safety and leads to operation shutdown, waste of resources, loss of product, reduced efficiency, increased maintenance needs, and cost over design^1–4^. Carbon steel is an ubiquitous alloy in many industrial fields such as metal processing equipment, petrochemical production, refining, and chemical processing owing to its low cost, ease of machinery, and unique mechanical properties compared to other metallic alloys^5^. Acid solutions are commonly utilized in chemical treatment processes such as acid cleaning, oil-well acidizing, acid descaling, etc. It is a highly aggressive medium for corrosion of carbon steel, as a result, such treatment with an acid brings about undesirable consequences, especially when used in direct contact on C-steel alloys^6^. One acceptable approach to combat corrosion is to incorporate corrosion inhibitors that reduce corrosion rates to the required level with minimal impact on the environment^7,8^. It is therefore, to date, the addition of corrosion inhibitors remains an essential procedure to mitigate the destructive attack of corrosive acid on the metal surface^9,10^. In a major number of studies, effective inhibitors are those organic compounds with hetero atoms such as Nitrogen, Oxygen, Sulfur, which show promising efficiency in mitigating the aggressive attack of corrosive species on metals^11,12^. These inhibitors act at the interface between the metal and the acidic solution and their interaction with the metal surface through the adsorption process that stops the dissolution of metal surface^13^. It is widely accepted that the adsorption mode of the inhibitor depends on some physiochemical properties of the molecules such as polarization of polar groups (O, N, P, S atoms, and π electrons), aromatic characterization, steric hindrance effects, electronic density, type of the corrosive environment, and nature of interactions between the π-orbital of inhibitors with the d-orbital of iron^14–17^. In literature, the selection of N-heterocyclic compounds is attributed to the presence of incorporated hetero-organic moieties which offer more protection potential against corrosion of steel. Carbonitrile derivatives are an interesting class of fused N-heterocyclic systems, which demonstrate a wide range of biological activities such as antimicrobial^18^, anticancer activities^19^, anti-inflammatory agents^20^, and act as effective inhibitors of metal corrosion^21,22^. Carbonitrile derivatives have been previously studied in literature for their inhibition efficiency against steel alloys^23–25^. Table 1 provides results from previous studies compared, including our work. However, we aim in our work to increase the efficiency of the studied inhibitors by synergetic effect of halides. To explore the performance of the PdC-OH derivative and explore the effect of the halide mixture, extensive research was carried out to identify the effect of I^−^ added to this derivative. The improvement of the efficiency of the organic inhibitor by halide ions is due to the bridge effect that occurs through the adsorption of halide ions on the surface of steel, which reduces its hydrophilicity and facilitates the adsorption of charged organic molecules^26^. The objective of this work is to study the corrosion inhibitory effect of 6-amino-4-aryl-2-oxo-1-phenyl-1,2-dihydropyridine-3,5-dicarbonitrile derivatives with and without the halide ion in 1 M HCl solution by some reliable methods by chemical and electrochemical measurements. Then, calculate standard thermodynamic and kinetic parameters and discussed the results to get an insightful overview of the active and adsorbed processes. In addition, the surface morphology of the C-steel surface was examined and discussed in detail. Furthermore, theoretical methods have been applied in our study to advance our understanding in many aspects of corrosion inhibition studies. The framework of quantum chemical calculations and Monte Carlo simulation method were performed to gain insight into the active centers of the investigated inhibitors other than the nature of the interaction between the metal surface and these inhibitors.

**Table 1:** %IE of some carbonitrile compounds in 1 M HCl and for steel corrosion.

Inhibitor	Material	Media	%IE	Reference
(i) 2-amino-7,7-dimethyl-10,30,5-trioxo-10,30,5,6,7,8-hexahydrospiro [chromene-4,20-indene]-3-carbonitrile (INH-1)	Mild steel	1 M HCl	89.35	^23^
(ii) 3-amino-7,7-dimethyl-20,5-dioxo-5,6,7,8-tetrahydrospiro[chromene-4,30-indoline]-2-carbonitrile (INH-2)			94.09	
(iii) 30-amino-70,70-dimethyl-2,50-dioxo-50,60,70,80-tetrahydro-2H-spiro [acenaphthylene-1,40-chromene]-20-carbonitrile (INH-3)			95.77	
3-methyl-6-oxo-4-(thiophen-2-yl)-4,5,6,7-tetrahydro-2hpyrazolo[3,4-b] pyridine-5-carbonitrile (TPP)	Mild steel	1 M HCl	95.75	^24^
The combined admixture of benzene carbonitrile and 5-bromovanillin (BNV 0.25%)	Carbon steel	1 M HCl	97.95	^25^
The combined admixture of benzene carbonitrile and 5-bromovanillin (BNV 0.25%)	Carbon steel	1 M HCl	97.95	^25^
(i) 6-Amino-2-oxo-1,4-diphenyl-1,2-dihydropyridine-3,5-dicarbonitrile (PdC-H)	Carbon steel	1 M HCl	80.5	Our work
(ii) 6-Amino-2-oxo-1-phenyl-4-(p-tolyl)-1,2-dihydropyridine-3,5-dicarbonitrile(PdC-Me)			78.5	
(iii) 6-Amino-4-(4-hydroxyphenyl)-2-oxo-1-phenyl-1,2-dihydropyridine-3,5-dicarbonitrile (PdC-OH)			77.8	

## Experimental methods

### Synthesis of inhibitors

**Figure 1:**
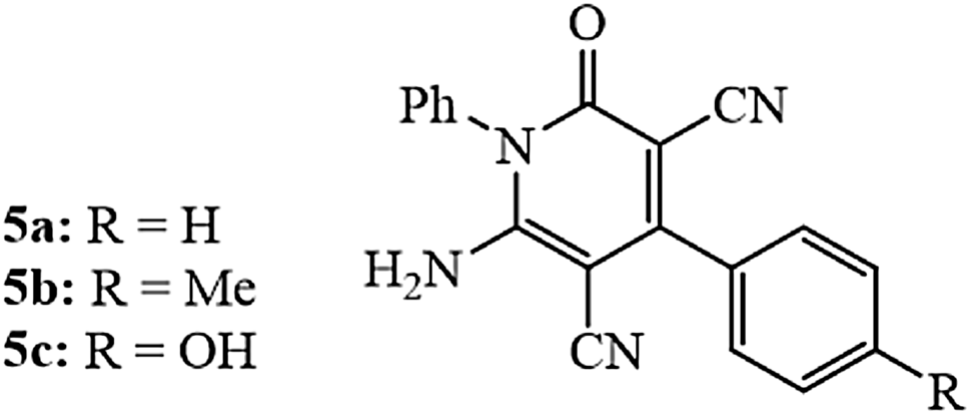
Molecular structure of investigated carbonitrile inhibitors.

The synthesis and full characterization of 6-amino-4-aryl-2-oxo-1-phenyl-1,2-dihydropyridine-3,5-dicarbonitrile derivatives (PdC-H, PdC-Me, and PdC-OH) with the structure in Table 2 were reported elsewhere^27^. Samples of synthesis batch were introduced to our lab for studying the efficiency of these compounds as new corrosion inhibitors. The molecular structures of the studied carbonitriles derivatives are shown in Fig. 1.

**Table 2:** Chemical structures of the tested compounds.

Inhibitor code	Molecular structures	Chemical name/mol. formulas
PdC-H		6-Amino-2-oxo-1,4-diphenyl-1,2-dihydropyridine-3,5-dicarbonitrile C_19_H_12_N_4_O
PdC-Me		6-Amino-2-oxo-1-phenyl-4-(p-tolyl)-1,2-dihydropyridine-3,5-dicarbonitrile C_20_H_14_N_4_O
PdC-OH		6-Amino-4-(4-hydroxyphenyl)-2-oxo-1-phenyl-1,2-dihydropyridine-3,5-dicarbonitrile C_19_H_12_N_4_O_2_

### Chemical treatment procedure

The experimental material is carbon steel with a chemical composition (weight%): Si 0.25%, C 0.2%, Mn 0.5%, S 0.05%, and balance Fe. Carbon steel specimens were mechanically cut into dimensions of 2.0 × 2.0 × 0.2 cm for weight loss and surface analysis experiments. Prior to all measurements, specimens were pretreated by abrading and polishing the surface to a mirror finish using successive grades of emery papers from 400 to 2000 grit size, degreased with acetone, washed with bi-distilled water, and dried at ambient temperature before weighing. A small carbon steel specimen was embedded in epoxy resin with an exposed surface area of 1 cm^2^ and used as a working electrode in the electrochemical experiments. Corrosion solution (1 M HCl) was prepared by diluting the analytical reagent grade 37% HCl with ultrapure distilled water. Finally, stock solutions of the investigated inhibitors (10^–3^ M) were prepared by using ethanol as a solvent. Inhibitor concentrations ranged ((2–10) × 10^–5^ M) by diluting the stock solutions with deionized water.

### Weight loss measurements

The weight-loss method is a high-accuracy method for laboratory corrosion study to determine the corrosion rate ($C.R$) and the inhibition efficiency (*η*). Pre-treatment and pre-weighed carbon steel specimens were immersed in the aggressive media in the absence and presence of the studied additives with the concentration range of ((2–10) × 10^–5^ M). Maximum immersion time was 180 min. After equal time intervals (30 min), specimens were taken from the acidic medium, rinsed with bi-distilled water, air dried, and precisely re-weighed. All measurements were performed in triplicate to ensure the reproducibility of the results. Average weight loss values ($\Delta W$) were recorded and the corrosion rate was calculated using equation^24^:

1 $$C.R=\frac{\Delta W}{\text{AT}}$$

where C.R is the corrosion rate (mg/cm^2^/min), $\Delta W$ is the average weight loss (mg) for the carbon steel specimens, $\text{A}$ is the surface area (cm^2^) and $\text{T}$ is the immersion duration. From the calculated $C.R$ values, the inhibition efficiency (*η*) can be calculated by the following equation^25^:

2 $${\%\upeta }=\frac{{CR}_{1}-{CR}_{2}}{{CR}_{1}} \times 100$$

where ${CR}_{1}$ and ${CR}_{2}$ are the corrosion rate values in the absence and presence of different concentrations of carbonitrile derivatives, respectively.

### Electrochemical measurements

Further corrosion tests were investigated electrochemically in a three-compartment glass cell comprising a working electrode (carbon steel electrode) with an uncovered area of 1 cm^2^, reference electrode (saturated calomel electrode), and counter electrode (platinum foil). Electrochemical measurements were performed under static conditions in a naturally aerated solution of 1 M HCl in the absence and presence of varying concentrations of three studied derivatives at 25 ± 1 °C. Before each electrochemical experiment, the system was maintained in an unperturbed state for 1800s to reach a stable value of the open circuit potential $\left(OCP\right)$. The polarization curves started from the cathodic direction to the anodic direction and were carried out by automatically sweeping the electrode potential from − 0.5 to + 0.5 V segment to $OCP$ at a scan rate of 0.5 mV s^−1^. Polarization parameters were obtained by extrapolation the anodic and cathodic regions of the Tafel plots. EIS measurements were performed after the attainment of steady-state $\text{OCP}$ by analyzing the frequency response of the electrochemical system with a range-extending from 0.01 Hz at low frequency to 100,000 Hz at high frequency and the excitation signal is a 5-mV sine wave. In the EFM technique a single, low-frequency, and low-distortion, sinusoidal voltage is applied to the corrosion interface (amplitude 10 mV with 2 and 5 Hz sine waves). Gamry PCI4-G750 Potentiostat/Galvanostat/ZRA. Echem Analyst V6.30 Software has been applied for fitting the electrochemical data.

### Surface analysis by ATR-IR, AFM and XPS

Morphological analyses methods were performed to evaluate the impact of corrosion and other characteristics of films on the surface of carbon steel. The mechanically prepared steel specimens were polished by emery paper in sequence to 2000 grade, degreased with absolute ethyl alcohol and acetone, washed with distilled water, and dried in a vacuum system. The pre-treated carbon steel surface is engrossed in 1 M HCl solution in the absence and presence of the optimal concentration of each studied derivative (10^−4^ M) at 25 °C for 24 h.

The chemical composition of the investigated compounds can be determined using ATR-IR (Thermo Fisher Scientific, NicoletiS10 model) analysis; Infrared spectra were recorded using ATR (Attenuated Total Reflection) in the wavenumber range 400–4000 cm^−1^. The morphological changes of the corroded carbon steel surface before and after treatment with the studied carbonitrile derivatives were assessed using non-contact mode atomic force microscopy (AFM) (Model: Thermo Fisher Nicolet IS10 (Scanning probe microscope)). The elemental composition and chemical states of the inhibitive film formed on the surface of carbon steel were examined by XPS (Model: Thermo Fisher Scientific) via Al Kα X-ray source (− 10 to 1350 eV) spot size 400 micro m at pressure 10–9 bar with full-spectrum pass energy 200 eV and at narrow-spectrum 50 eV (produced in USA K-ALPHA).

### Quantum chemical calculations

To inspect the correlation between the molecular structure and the reactivity of 6-amino-4-aryl-2-oxo-1-phenyl-1,2-dihydropyridine-3,5-dicarbonitrile derivatives, theoretical calculations were performed using the DMol3 module adopted in Materials Studio version 7.0. Within the DMol3 module, a basis set of double number polarization (DNP) plus the exchange–correlation functions of Becke One Parameter (BOP) with generalized gradient approximation (GGA) and solvent effects were treated using COSMO controls^26^. Quantum calculations are also performed using Orca 4.1, and the calculation inputs are set at the DFT level with DEF2-SVP as basis Set and B3LYP as functional. The aim of using two different methods and software is to verify the consistency between the experimental and theoretical results in anticipation of the ranking of inhibition efficiency of studied compounds^6^.

### Monte Carlo simulations

MC simulations were performed to explore the interaction between the inhibitor molecules and the iron surface. The adsorption simulation of the compound on the carbon steel surface in HCl medium was performed using the module of adsorption locator applied in Materials Studio 2107. Carbon steel is significantly made of iron atoms, so the surface was obtained by the cleaved plane of iron identified as the stable crystal plane for iron. The optimized structures of the inhibitor molecules from the DFT study were used in Monte Carlo Simulations. The simulation proved to be related to the experimental study, in which the adsorption of each inhibitor molecule on Fe (1 1 0) was simulated in the presence of hydrochloric acid solution characterized by H_3_O^+^ and Cl^−^ ions in the abundance of water molecules. The simulation was carried out by Monte Carlo method^27–29^.

## Results and discussion

### Weight loss method

#### Effect of concentrations

**Figure 2:**
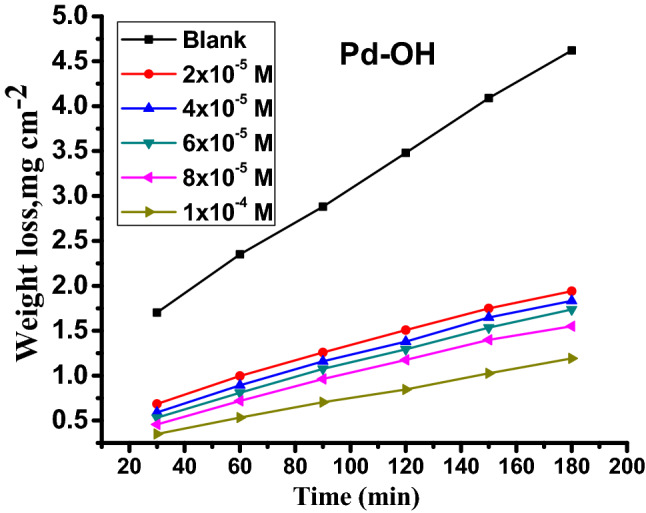
Weight loss-time curve for the dissolution of carbon steel in 1 M HCl without and with various concentrations of PdC-OH at 25 °C.

To assess the effect of varying concentrations of carbonitrile derivatives ((2–10) × 10^–5^ M) on the effectiveness of corrosion inhibition, weight loss measurements were performed in 1 M HCl solution. Figure 2 presents the weight loss-time curve for carbon steel in 1 M HCl in the absence and presence of different concentrations of PdC –OH at 25 °C. The weight loss-time curves for PdC-Me, PdC-H are found in the supplementary material (Fig. S1). It is evident that the weight loss of carbon steel in the inhibitor-containing solutions decreases over time with increasing the concentration of inhibitors^30^. The corrosion rate $\left(C.R\right)$, the degree of the surface coverage $\left(\theta \right)$, and the inhibition efficiency (%*η*) are summarized in Table 3. These results indicate that the addition of the studied derivatives in the acidic medium reduced the corrosion rate, which led to an increase in the degree of surface coverage of the inhibitor on the steel surface. In addition, the inhibition efficiency %*η* increases as the inhibitor concentration increases indicating the formation of an adsorbed barrier layer upon the steel surface, wherein the inhibitor acts as an adsorbate and the metal surface behaves as an adsorbent. Interestingly, the PdC –OH compound shows higher values of %η than PdC-Me and PdC-H, signifying that the molecular structure of the inhibitors affected the adsorption properties of organic molecules on the steel surface. The strong conjugation between benzene and the pyridine ring and the specific hetero-aromatic ring (–OH) promotes the adsorption of the derivative on the steel surface, thus increasing %*η*^31^.

**Table 3:** Data of Wt. loss method for corrosion of carbon steel in 1 M HCl solution with and without various concentrations of PdC-OH, PdC-Me and PdC-H compounds at 25 °C ± 0.1.

Compounds	Conc. M	C.R (mg/cm^2^/min)	θ	%*η*
1 M HCl	Blank	0.029 ± 0.0023	–	–
PdC-OH	2 × 10^–5^	0.012 ± 0.0015	0.586	58.6
	4 × 10^–5^	0.011** ± **0.0012	0.604	60.4
	6 × 10^–5^	0.011 ± 0.0018	0.619	61.9
	8 × 10^–5^	0.010 ± 0.0009	0.662	66.2
	1 × 10^–4^	0.007 ± 0.0029	0.757	75.7
PdC-Me	2 × 10^–5^	0.017** ± **0.0020	0.425	42.5
	4 × 10^–5^	0.014 ± 0.0009	0.526	52.6
	6 × 10^–5^	0.012 ± 0.0023	0.575	57.5
	8 × 10^–5^	0.011 ± 0.0017	0.615	61.5
	1 × 10^–4^	0.008 ± 0.0020	0.727	72.7
PdC-H	2 × 10^–5^	0.018** ± **0.0017	0.397	40.0
	4 × 10^–5^	0.014 ± 0.0015	0.515	51.5
	6 × 10^–5^	0.014 ± 0.0026	0.525	52.5
	8 × 10^–5^	0.012 ± 0.0026	0.598	59.8
	1 × 10^–4^	0.009 ± 0.0023	0.704	70.4

#### The effect of KI added to PdC-OH compound

**Figure 3:**
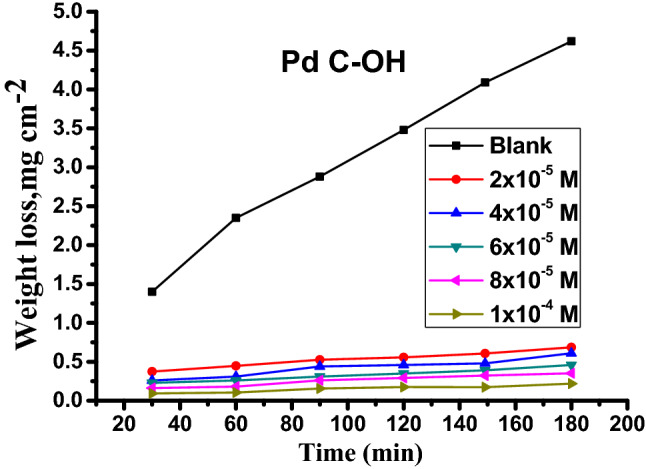
Weight loss-time curve for dissolution of carbon steel in 1 M HCl without and with various concentrations of PdC-OH + KI at 25 °C.

Adding KI can increase the inhibition efficiency as cited^32^. The addition of KI to PdC-OH results in higher inhibition efficiency. As indicated from Table 4, the inhibition efficiency of individual PdC-OH at 10^–4^ M is 75.7% (from weight loss results), while the inhibition efficiency of PdC-OH in combination with KI is 91.67%. In the presence of the auxiliary inhibitor and KI, the inhibition efficiencies increase with increasing the concentration, which can be shown from the curves in Fig. 3. The combination of PdC-OH and KI shows a synergistic effect. The synergism parameter S_θ_ is calculated as follows^33^:

**Table 4:** Data of Wt. loss method for corrosion of carbon steel in 1 M HCl solution with and without KI + PdC-OH (M) at 25 °C ± 0.1.

Compound	Conc. M	$C.R$ (mg/cm^2^/min)	θ	%*η*
1 M HCl	Blank	0.029 ± 0.0038	–	–
PdC-OH + KI	2 × 10^–5^	0.004** ± **0.00018	0.851	85.06
	4 × 10^–5^	0.004 ± 0.0002	0.868	86.78
	6 × 10^–5^	0.003 ± 0.0003	0.899	89.94
	8 × 10^–5^	0.003** ± **0.0001	0.905	90.52
	1 × 10^–4^	0.002** ± **0.0004	0.917	91.67

3 $${S}_{\theta }=1-({\theta }_{1+2})/1-{\theta }_{1+2}^{^{\prime}}$$

where ${\theta }_{1+2}=\left({\theta }_{1}+{\theta }_{2}\right)-({\theta }_{1}*{\theta }_{2})$ $\theta$_1_ is the surface coverage by the main inhibitor; $\theta$_2_ is the surface coverage by the auxiliary inhibitor; $\theta$′_1+2_ is the measured surface coverage by both the main inhibitor and the auxiliary inhibitor (the concentration of the main inhibitor 1 (PdC-OH in this case) and the auxiliary inhibitor 2 (KI in this case) in the mixture should be the same as used responding to separate situations). If S_θ_ approaches 1, it indicates that there are no interactions between the two inhibitors, while if S_θ_ > 1, the synergistic effect exists; in the case of S_θ_ < 1, the antagonistic interaction might prevail. The value of the synergism parameter for PdC-OH and KI at 6 × 10^−5^ M studied from weight loss measurement is 1.2 and the value of the synergism parameter for PdC-OH and KI at 8 × 10^–5^ is 1.21 both are larger than 1. This synergistic process occurs through the oxidation of I^–^ ions in the solution by the dissolved oxygen, resulting in the generation of I_2_. Then ${I}_{2}$ combine with ${I}^{-}$ to form soluble yellowish^33^ which acts as a bridge and connects between the inhibitor and the steel surface. The protection of the steel surface and thereby greater inhibition ability is mainly contributed to the presence of the main inhibitor (PdC-OH) and the existence of the adsorbed iodide ions.

### Adsorption isotherm

**Figure 4:**
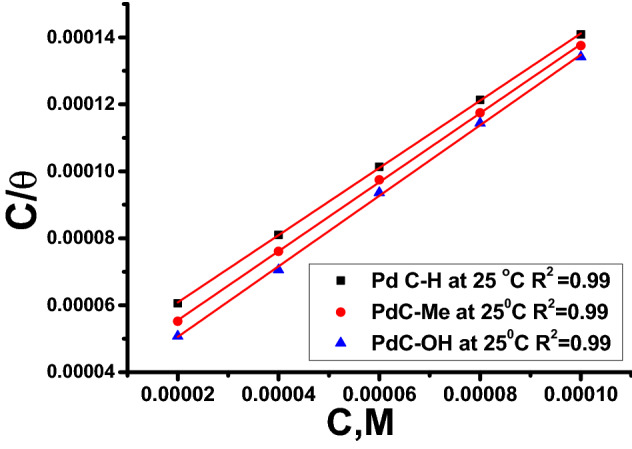
Langmuir isotherms draw as [C/θ] vs. [C] of (PdC-H, PdC-Me and PdC-OH) for corrosion of carbon steel in 1 M HCl at 25 °C.

The performance of the inhibitor molecules in the aqueous medium results from their adsorption propensity on the corroded surface of the metal and interfering with the electrochemical reactions over the area covered by the inhibitor molecule. Several isotherms equations such as Frumkin, Langmuir, Temkin, and Freundlich were deduced by fit of the surface coverage ($\theta$) from experimental data as a function of concentration ($c$) to determine the nature of interactions between the investigated organic molecules and the corroding metal surface during the corrosion inhibition process. The Langmuir isotherm equation is the best-fit equation to the results where the values of the regression coefficient $\left({R}^{2}\right)$ approaches from unity. Langmuir Isotherm Plots is provided in Fig. 4. This behavior indicates that a monolayer of the adsorbed inhibitor was formed on the surface of the metal substrate according to the Langmuir equation (4)^34–37^:

4 $$\frac{C}{\theta }=\left(\frac{1}{{K}_{ads}}\right)+c$$

As shown in Fig. 4, C/θ is the ordinate (Y-axis), C is the abscissa (X-axis), 1/*K*_*ads*_ is the intercept where the equilibrium constant ${(k}_{ads})$ values can be determined from this interception. Furthermore, the standard adsorption Gibbs free energy ($\Delta {\text{G}}_{ads}^{o}$), enthalpy ($\Delta {\text{H}}_{ads}^{o}$) and entropy ($\Delta {\text{S}}_{ads}^{o}$) of adsorption can be assessed using the following Eqs. (5), (6) and (7), respectively^38–40^:

5 $${K}_{ads}=\frac{1}{55.5} \times exp\left(\frac{{{- \Delta \text{G}}^{o}}_{ads}}{RT}\right)$$

6 $${\ln} \, {K}_{ads}= \frac{-\Delta {\text{H}}^{o}}{RT}+constant$$

7 $$\Delta {\text{G}}_{ads}^{o}=\Delta { \text{H}}_{ads}^{0}-\Delta {\text{S}}_{ads}^{o}$$

All the above-calculated adsorption parameters are mentioned in Table 4. The negative values of ${\Delta {\text{G}}^{o}}_{ads}$ approves the spontaneous of the adsorption process and strong interactions between the inhibitor molecules and the metal surface. Besides, the high values of ${k}_{ads}$ revealed an effective adsorption process with high efficiency of the inhibitor molecules^41^. From Table 4, the absolute values of ${\Delta {\text{G}}^{o}}_{ads}$ of the three inhibitors were − 35.7 and − 35.4, − 35.3 kJ mol^−1^ respectively. Commonly, the values of ${ \Delta {\text{G}}^{o}}_{ads}$ are less negative than − 20 kJ mol^−1^ which belongs to physisorption (electrostatic interaction) between the inhibitor molecules and the metal surface. Conversely, negative values than − 40 kJ mol^−1^ indicate chemical adsorption, which is owed to adsorption of organic molecules on the metal surface accompanied by charge transfer to form chemical bonds. As above mentioned, the free energy of all studied inhibitors was in the range of − 20 kJ mol^−1^ to − 40 kJ mol^−1^, indicating that the type of adsorption process belongs to the mixed adsorption type (including physisorption and chemisorption)^42,43^. Also as shown in Table 5, the values of ${\Delta {\text{G}}^{o}}_{ads}$ were very close to − 40 kJ mol^−1^ and increased with temperature from (25 to 40) ºC which was attributed to the adsorption of carbonitrile derivatives on the steel surface dependent mainly on chemical absorption^44^. In the literature, the enthalpy of adsorption ($\Delta {\text{H}}_{ads}^{0}$> 0) and close to (100 kJ mol^−1^) reflects the endothermic behavior of the adsorption. While it is exothermic at ($\Delta {\text{H}}_{ads}^{0}$< 0), the enthalpy less (40 kJ mol^−1^) may involve either physisorption, or chemisorption, or a combination of both^45^. As seen in the current study, the calculated enthalpy $\Delta {\text{H}}_{ads}^{0}$ values have positive signs and less (40 kJ mol^−1^), indicating that the endothermic nature of the adsorption process and two types of adsorption interactions are present on the steel surface. The values of $\Delta {\text{S}}_{ads}^{o}$ are increased in a positive direction which is mainly typical of the endothermic adsorption process that is equivocally related to chemical adsorption. This increase can be attributed to the increased randomness due to the formation of the adsorbed layer of the inhibitors and the desorption of a high number of water molecules at the carbon steel/solution interface^46^.

**Table 5:** Thermodynamic adsorption parameters of (PdC-OH, PdC-Me and PdC-H) adsorbed on the carbon steel surface in 1 M HCl acid at various temperatures.

Inhibitor	Temp °C	${K}_{ads}$ M^−1^	$-{\Delta G}_{ads}$ kJ mol^−1^	$\Delta {\text{H}}_{ads}^{0}$ kJ mol^−1^	$\Delta {\text{S}}_{ads}^{o}$ J mol^−1^ K^−1^
PdC-OH	25	32,809.5	35.7 ± 0.2028	4.1	119.9 ± 0.2028
	30	36,934.9	36.6 ± 0.1732		120.9 ± 0.2333
	35	40,524.1	37.4 ± 0.1453		121.6 ± 0.1453
	40	53,827.7	38.8 ± 0.2028		123.9 ± 0.1453
PdC-Me	25	29,344.9	35.4 ± 0.1028	5.1	118.9 ± 0.1732
	30	36,488.6	36.5 ± 0.1732		120.8 ± 0.1453
	35	40,555.4	37.5 ± 0.2028		121.6 ± 0.2028
	40	67,268.9	39.5 ± 0.1453		125.8 ± 0.1764
PdC-H	25	25,750.7	35.3 ± 0.1732	4.2	117.9 ± 0.1453
	30	33,662.9	36.5 ± 0.2028		120.1 ± 0.1732
	35	33,759.1	37.3 ± 0.1732		120.1 ± 0.2028
	40	46,040.1	38.7 ± 0.1000		122. 7 ± 0.2309

### Effect of temperature

**Figure 5:**
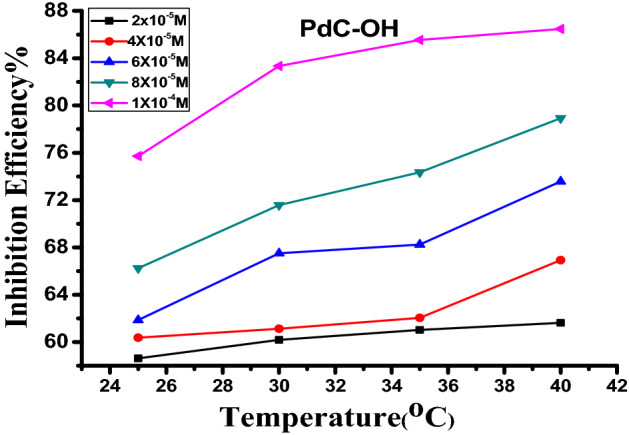
Difference of %η against different solution temperatures for compound PdC-OH.

Temperature is a predominant factor in the corrosion inhibition process, in which corrosion is accelerated at elevating the solution temperature and affects the action of corrosion inhibitors. To investigate the effect of temperature on the dissolution of carbon steel in the presence and absence of different concentrations of carbonitrile derivatives, weight loss measurements were performed at varying temperatures ranged (25–40 °C). The variation of %*η* is plotted vs. the above-mentioned temperatures for PdC-OH derivative in Fig. 5, similar plots for PdC-Me and PdC-H are found in the supplementary material (Fig. S2). The corrosion rate (C.R) is obviously exaggerated and the inhibition efficiency increases with increasing solution temperature^43^. This action indicated that the investigated inhibitor molecules adsorbed on the steel/solution interface^30^. Moreover, the slight increase or constancy in the inhibition efficiency with increasing temperatures is attributed to the chemical adsorption of the inhibitor species only or is owed to a combination of chemical and physical adsorptions^47^.

### Thermodynamic kinetic parameters

**Figure 6:**
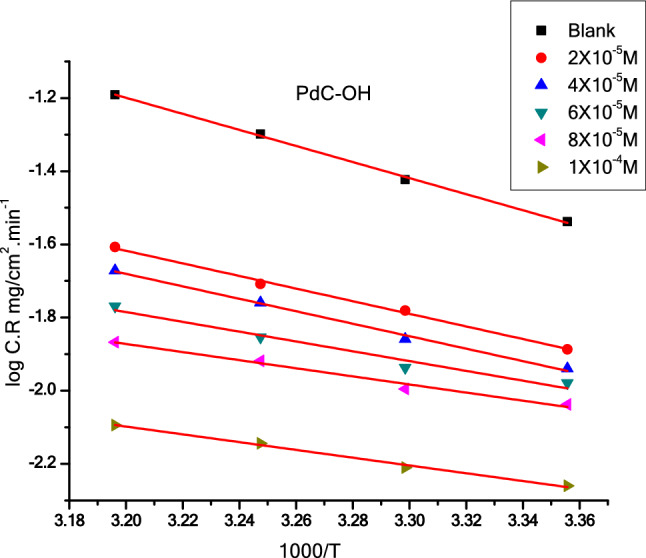
Arrhenius plot (log C.R vs 1000/T) for carbon steel in 1 M HCl with and without various concentrations of PdC-OH compound.

To get more insights on the mechanism of the adsorption process of inhibitor molecules on the surface of the metallic material, the value of apparent activation energy ${E}_{a}^{*}$ is frequently utilized to determine the type of adsorption mechanism. It can be calculated from the plot of the Arrhenius equation shown in Fig. 6, The Arrhenius plots of PdC-Me and PdC-H are found in the supplementary material (Fig. S3). Various parameters of the corrosion process based on Arrhenius equation (8) and the transition state theory equation (9) presented in the following equations^48^:

8 $${\log} \, C.R={\log} \, A-\left(\frac{{E}_{a}^{*}}{2.303RT}\right)$$

9 $$C.R=\left(\frac{RT}{Nh}\right){e}^{\left(\frac{\Delta {\text{S}}^{*}}{R}\right)}{e}^{\left(\frac{\Delta {\text{H}}^{*}}{RT}\right)}$$

where A is the pre-exponential factor (Arrhenius constant), $\Delta {\text{H}}^{*}$ is the activation enthalpy, $\Delta {\text{S}}^{*}$ is the activation entropy, T is the absolute temperature in Kelvin, h is the Plank constant, N is the Avogadro number and R is the molar gas constant. As depicted in Table 6, the value of ${E}_{a}^{*}$ in the blank solution is relatively higher than that in the presence of three inhibitors; this is ascribed to the phenomenon of chemical adsorption, whereas the opposite is true for physical adsorption^49^. The positive values of activation enthalpy ($\Delta {\text{H}}^{*}$) indicate that the activation process is an endothermic corrosion process^41^. This phenomenon indicates that high temperature accelerates the corrosion process of carbon steel, and thus is attributed to chemical adsorption. The change in activation entropy $\Delta {\text{S}}^{*}$ increases in a positive direction inferring that the activated complex in the transition state is formed by association rather than dissociation process. In other words, more ordering will occur when the reactants convert to the active complex^50^.

**Table 6:** Activation parameters for the corrosion of carbon steel in the absence and presence various molar concentrations of PdC-OH, PdC-Me and PdC-H compounds in 1 M HCl.

Inhibitor	Conc., M	Activation parameters		
		${E}_{a}^{*}$	$\Delta {\text{H}}^{*}$	− $\Delta{\text{ S}}^{*}$
		kJ mol^−1^	kJ mol^−1^	J mol^−1^ K^−1^
Free acid (1 M HCl)		42.2 ± 0.2309	21.2 ± 0.2309	111.4 ± 0.2404
PdC-OH	2 × 10^–5^	35.7 ± 0.2028	13.2 ± 0.2603	179.0 ± 0.1528
	4 × 10^–5^	33.6 ± 0.2603	12.8 ± 0.1732	183.2 ± 0.2333
	6 × 10^–5^	25.0 ± 0.2333	11.5 ± 0.2603	195.2 ± 0.2309
	8 × 10^–5^	21.2 ± 0.2603	11.4 ± 0.1552	196.8 ± 0.2729
	1 × 10^–4^	20.3 ± 0.1712	11.8 ± 0.1642	195.8 ± 0.1471
PdC-Me	2 × 10^–5^	36.0 ± 0.1522	14.8 ± 0.1712	165.0 ± 0.1463
	4 × 10^–5^	33.3 ± 0.1602	13.1 ± 0.2313	179.1 ± 0.1528
	6 × 10^–5^	31.9 ± 0.1422	12.3 ± 0.2404	186.1 ± 0.1764
	8 × 10^–5^	29.9 ± 0.1852	11.7 ± 0.1453	191.8 ± 0.1732
	1 × 10^–4^	27.5 ± 0.1312	11.8 ± 0.2025	194.0 ± 0.1764
PdC-H	2 × 10^–5^	37.7 ± 0.1528	15.1 ± 0.2138	162.4 ± 0.1856
	4 × 10^–5^	36.8 ± 0.2028	13.9 ± 0.2426	172.6 ± 0.1764
	6 × 10^–5^	35.2 ± 0.2603	12.6 ± 0.2125	183.1 ± 0.1528
	8 × 10^–5^	30.4 ± 0.2048	11.7 ± 0.2344	191.8 ± 0.1453
	1 × 10^–4^	26.9 ± 0.2348	11.5 ± 0.2033	195.8 ± 0.2646

### Potentiodynamic polarization technique

**Figure 7:**
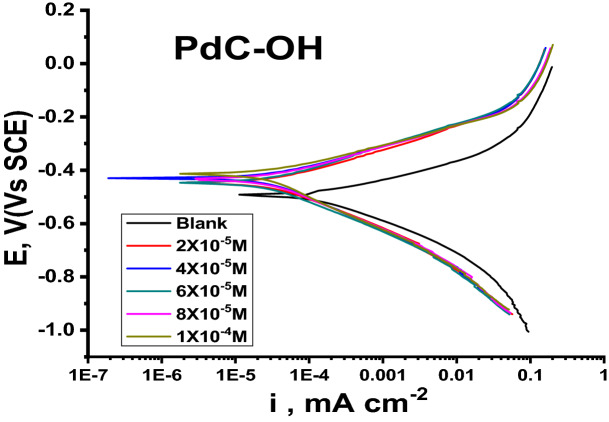
Potentiodynamic polarization plot for the corrosion of carbon steel in 1 M HCl without and with different concentrations of PdC-OH at 25 °C.

Polarization measurements were performed to understand the nature of electrochemical kinetics reactions. Figure 7 shows the polarization behavior of the carbon steel electrode in 1 M HCl in the absence and presence of various concentrations of the PdC-OH compound. The current potential plots of PdC-Me and PdC-H are found in the supplementary material (Fig. S4). Relevant electrochemical kinetic parameters such as corrosion current density (${I}_{corr}$) and cathodic $\left({\beta }_{c}\right)$ anodic $\left({\beta }_{a}\right)$ Tafel slopes were obtained from the polarization curves by extrapolating Tafel lines with respect to the corrosion potential ${E}_{corr}$^51^. Equation (10) represents the correlation between inhibition efficiency and corrosion current density is represented as follows:

10 $${\%}\eta =\frac{{I}_{corr}^{o}-{I}_{corr}^{i}}{{I}_{corr}^{o}}$$

where ${I}_{corr}^{o}$ and ${I}_{corr}^{i}$ represent the corrosion current densities of the uninhibited and inhibited solutions, respectively. All these parameters are tabulated in Table 7.

**Table 7:** Electrochemical parameters from polarization measurements for carbon steel corrosion in 1 M HCl for PdC-OH, PdC-Me and PdC-H derivatives at 25 °C.

Conc, M		i_corr._ mA cm^−2^	− E_corr,_ mV vs.SCE	β_a,_ mV dec^−1^	− β_c,_ mV dec^−1^	C.R (mm y^−1^)	θ	%*η*
Blank		150.0	493 ± 0.2333	71.0 ± 0.2309	122.2 ± 0.2028	98.1	**–**	**–**
PdC-OH	2 × 10^–5^	35.0 ± 0.1155	448 ± 0.2028	92.9 ± 0.2309	139.8 ± 0.3528	14.5	0.767	76.7
	4 × 10^–5^	32.3 ± 0.1732	433 ± 0.1453	81.3 ± 0.1453	133.7 ± 0.2028	13.4	0.785	78.5
	6 × 10^–5^	31.6 ± 0.2603	430 ± 0.1453	79.7 ± 0.1732	134.7 ± 0.1453	13.1	0.789	78.9
	8 × 10^–5^	30.6 ± 0.1764	398 ± 0.1732	65.8 ± 0.2309	151.1 ± 0.2906	12.7	0.796	79.6
	1 × 10^–4^	29.2 ± 0.1732	414 ± 0.1732	71.4 ± 0.2028	143.0 ± 0.2028	12.1	0.805	80.5
PdC-Me	2 × 10^–5^	40.4 ± 0.2028	424 ± 0.1453	81.5 ± 0.1732	151.4 ± 0.1763	16.7	0.731	73.1
	4 × 10^–5^	38.2 ± 0.1732	433 ± 0.2082	85.0 ± 0.2309	150.9 ± 0.1732	15.8	0.745	74.5
	6 × 10^–5^	33.7 ± 0.2028	392 ± 0.1732	72.8 ± 0.1202	160.0 ± 0.1155	13.9	0.775	77.5
	8 × 10^–5^	33.4 ± 0.2309	442 ± 0.1522	87.0 ± 0.2333	145.4 ± 0.2028	13.8	0.777	77.7
	1 × 10^–4^	32.3 ± 0.1732	403 ± 0.1652	72.4 ± 0.1453	154.6 ± 0.1764	13.4	0.785	78.5
PdC-H	2 × 10^–5^	42.2 ± 0.1453	429 ± 0.1302	82.6 ± 0.2603	148.3 ± 0.2028	17.5	0.719	71.9
	4 × 10^–5^	40.0 ± 0.2603	420 ± 0.1135	80.7 ± 0.1732	152.0 ± 0.1265	16.6	0.733	73.3
	6 × 10^–5^	36.4 ± 0.2524	439 ± 0.2082	83.0 ± 0.1732	141.8 ± 0.1557	15.1	0.757	75.7
	8 × 10^–5^	34.5 ± 0.1453	452 ± 0.2309	86.60.1453	137.5 ± 0.1058	14.3	0.770	77.0
	1 × 10^–4^	33.5 ± 0.1202	391 ± 0.1453	74.5 ± 0.2082	153.3 ± 0.1741	13.9	0.778	77.8

As can be seen in Fig. 7, the cathodic and anodic Tafel lines are parallel upon adding these derivatives into acidic solution relative to the blank sample and have no substantial changes with each other. Thus, the adsorbed inhibitor merely hinders the active site of the anodic and cathodic reactions on the metal surface without affecting the actual corrosion mechanism, and only causes inactivation of part of the surface with respect to the corrosive medium^52^. Inspection the data in Table 7, the slopes of the anodic $\left({\beta }_{a}\right)$ and cathodic $\left({\beta }_{c}\right)$ Tafel lines slightly changed upon addition of these derivatives. It could be argued that these organic derivatives have the function of controlling the activation of hydrogen evolution and the anodic dissolution of the metal without any variation in the dissolution technique^53^. Another finding from Table 6 is that the ${E}_{corr}$ values for the inhibitory systems shifted to positive potential with the change in inhibitor concentrations less than 85 mV. This observation indicated that the studied derivatives behaved as mixed-type inhibitors and affect both the cathodic and anodic polarization curves^54^. As well, the maximum shift in the corrosion potential ${E}_{corr}$ with respect to ${E}_{corr}$ (blank) is more than 85 mV (observed for PdC-OH at 8 × 10^–5^ M, PdC-Me at 6 × 10^–5^ M, and PdC-H at 1 × 10^–4^ M). This attributed to that the three derivatives can be classified as cathodic or anodic inhibitors according to the previous literature^55^. The decrease in the corrosion current density $\left({i}_{corr}\right)$ with the incremental concentrations of the investigated inhibitors leads to an increase in the inhibition efficiency. This phenomenon pronounced that the investigated inhibitors adsorbed on the active sites and formed a more stable layer on the surface of carbon steel^56^. As summarized in Table 7, the %*η* order is followed as PdC-OH > PdC-Me > PdC-H. However, the difference in %η between the three compounds was small which can be attributed to the similar structures of the three derivatives.

### Electrochemical impedance spectroscopy (EIS)

**Figure 8:**
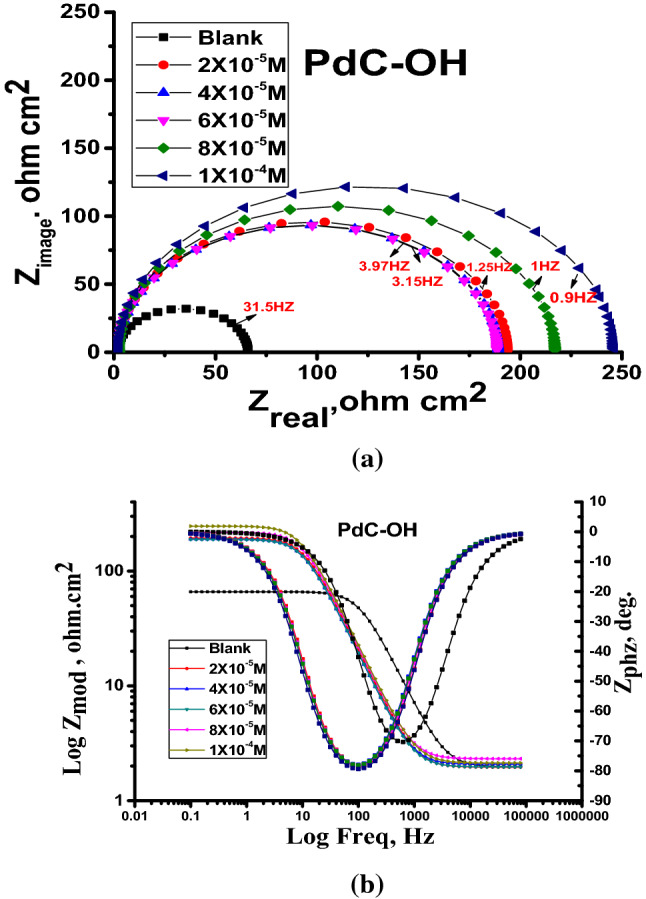
Nyquist (**a**) and Bode-phase angle (**b**) plots for corrosion of carbon steel in 1 M HCl in the absence and presence of various concentrations of PdC-OH at 25 °C.

**Figure 9:**
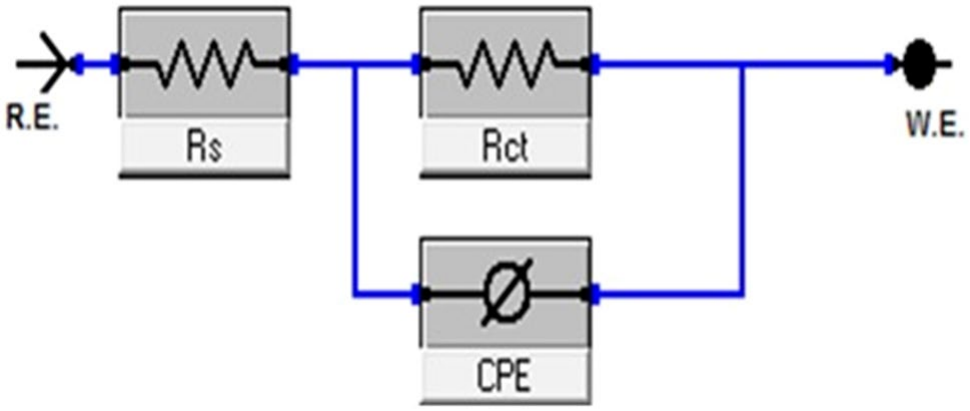
Equivalent circuit model used to fit the impedance spectra.

The EIS or AC impedance technique provides important mechanical and kinetic information for the electrochemical system under study. Impedance measurements were utilized to evaluate the corrosion resistance of the carbon steel electrode in 1 M HCl solution in the absence and presence of different concentrations of the investigated derivatives at 25 °C. The impedance spectra include (Nyquist and Bode) plots for PdC-OH are shown in Fig. 8a,b, Nyquist and Bode plots of PdC-Me, and PdC-H are found in the supplementary material (Figs. S5 and S6). Obviously, In the Nyquist plot, the appearance of an individual capacitive loop is represented as a slightly depressed semi-circle. This capacitive loop indicates a non-ideal capacitor performance at the metal/solution boundary phase^57,58^. Besides, the diameters of these capacitive loops increase significantly when the concentration of inhibitors in the test solution is increased without affecting their characteristic features. This is an indication that the adsorption of the studied inhibitors retards the corrosion of carbon steel in 1 M HCl without altering the electrochemistry of the corrosion process^59^. Moreover, for all tested inhibitors, the Bode-Phase plots showed a single peak within the studied frequency range, revealing that the impedance measurements were fitted in a one-time constant equivalent model with $CPE$. Furthermore, there is only one phase maximum in Bode plots which relates only to one relaxation process (one time constant). This may be attributed to the charge transfer process that occurs at the metal–electrolyte interface. The increase of the impedance modulus and the gradual increase of the phase angle maxima at the intermediate frequency with increasing concentration of the inhibitor were also shown^60^. This is due to more molecules being adsorbed on the surface of the electrode with increasing concentration and forming a protective layer as a barrier to the dissolution of the metal in acidic solutions. Besides, the phase angle values around 80, this deviation from the ideal corrosive system (phase angle = 90) is ascribed to the surface roughness as a result of both structural and interfacial origin^61^ For a more in-depth understanding of the impedance spectra; A simple electrical equivalent circuit in Fig. 9 was employed to fit these experimental spectra^62^. The circuit consists of constant phase element $\left(CPE\right)$, charge transfer resistor $\left({R}_{ct}\right) ,$ and solution resistance $\left({R}_{s}\right)$. Inhibition efficiency (*η*%) is calculated from ${R}_{ct}$ values using the following formula Eq. (11)^58^:

11 $${\%\upeta }=\frac{{R}_{ct}-{R}_{ct}^{0}}{{R}_{ct}}$$

where ${R}_{ct}^{0}$, ${R}_{ct}$ is the charge transfer resistance without and with inhibitor, respectively.

However, $${R}_{ct}$$ is a measure of electron transfer at electrode/electrolyte interface, which is inversely proportional to the corrosion rate^59^. Since the metal solution interface behaves as a double layer but without ideal capacitive behavior. Thus, the constant phase element $$\left(CPE\right)$$ was used to improve the capacitance of the electrical double layer instead of utilizing the absolute capacitance double layer ($${C}_{dl})$$. The impedance of the $$CPE$$ $$\left({Z}_{CPE}\right)$$ can be calculated using Eq. (12)^58^:

12 $${z}_{CPE={Y}_{0}^{-1} \left(j\varpi\right)}-n$$

where ${\text{Y}}_{\text{O}}$ is the magnitude of the $\text{CPE}$, ${\varpi}$ signifies the angular frequency $\left(\varpi=2{\uppi} \text{f}\right)$, n is the CPE exponent which depends on the nature of the metallic surface representing the deviation from the perfect capacitive performance that value is between 0 and 1. $\text{j}={(-1) }^{1/2}$ represents an imaginary number. The capacitance double layer (${\text{C}}_{\text{dl}})$ values of ${\text{Y}}_{\text{O}}$ and n are calculated as follows Eq. (13)^58^:

13 $${\text{C}}_{\text{dl}}={\text{Y}}_{\text{O}}\left({\varpi}_{\text{max}}\right)_{\text{n}-1}$$

where $\varpi$ is the angular frequency when the imaginary component of the impedance is at its maximum value. The preceding impedance spectroscopy parameters were listed in Table 8, and it is worth mentioning that the values of ${R}_{ct}$ increased with increasing the concentration of the inhibitor and consequently the inhibition efficiency increased *η*%. This is an evidence of the effective corrosion protection of steel in the presence of the studied compounds. The dramatic drop in ${\text{C}}_{\text{dl}}$ values upon addition of the inhibitor is owed to the replacement of the water molecules by the inhibitor molecules adsorbed at the interface. Hence, the formation of an adherent film on the metal surface leads to the increase in the thickness of the electric double layer and /or the reduction in the local dielectric constant at the metal/solution interface^50^. These outcomes approve that carbonitrile derivatives exhibit good carbon steel inhibitory properties in acidic solutions. Furthermore, the corrosion inhibition efficiencies calculated from electrochemical impedance spectroscopy measurements presented in Table 8 shows an agreement trend with the calculated data from weight loss experiments and potentiodynamic polarization measurements.

**Table 8:** Electrochemical kinetic parameters obtained from EIS technique for the corrosion of carbon steel in 1 M HCl at various concentrations of PdC-OH, PdC-Me and PdC-H at 25 °C.

[Inh]	Conc, M	R_ct_, Ω cm^2^	C_dl_x10^–5^, µFcm^−2^	Θ	%*η*
Free acid 1.0 M HCl	Blank	66.7 ± 0.1453	2.7600	–	–
PdC-OH	2 × 10^–5^	250.0 ± 0.2333	0.7080	0.733	73.3
	4 × 10^–5^	257.3 ± 0.17634	0.6880	0.741	74.1
	6 × 10^–5^	273.9 ± 0.1453	0.6320	0.757	75.7
	8 × 10^–5^	282.1 ± 0.1732	0.5230	0.764	76.4
	1 × 10^–4^	304.8 ± 0.1453	0.4350	0.781	78.1
PdC-Me	2 × 10^–5^	197.0 ± 0.2333	1.3500	0.661	66.1
	4 × 10^–5^	221.9 ± 0.2573	1.0300	0.699	69.9
	6 × 10^–5^	248.5 ± 0.2135	0.8220	0.732	73.2
	8 × 10^–5^	251.2 ± 0.2013	0.7920	0.734	73.4
	1 × 10^–4^	258.4 ± 0.2028	0.6850	0.742	74.2
PdC-H	2 × 10^–5^	175.6 ± 0.1732	1.6500	0.620	62.0
	4 × 10^–5^	184.2 ± 0.1512	1.5000	0.638	63.8
	6 × 10^–5^	191.2 ± 0.1422	1.3900	0.651	65.1
	8 × 10^–5^	201.8 ± 0.1352	1.2100	0.670	67.0
	1 × 10^–4^	218.7 ± 0.1732	0.9100	0.695	69.5

### Electrochemical frequency modulation

**Figure 10:**
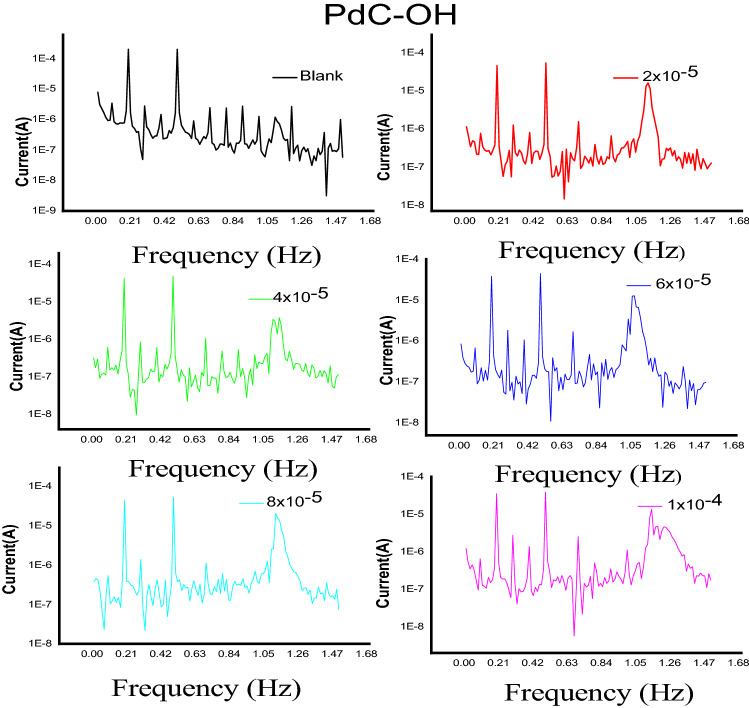
EFM spectra of carbon steel in 1 M HCl without and with various concentrations of PdC-OH at 25 °C.

Electrochemical frequency modulation technique is a powerful tool for monitoring metal corrosion in aqueous solutions. The foremost advantages of EFM are its rabid and non-destructive properties when applied to the corroded electrode. EFM has a substantial feature since the corrosion current can be determined from small polarization and AC signals without prior knowledge of the Tafel constants^63^. Intermodulation spectra acquired from the EFM technique for evaluating different concentrations of the studied derivative (PdC-OH) versus corrosive media 1 M HCl on the steel electrode are characterized in Fig. 10, EFM spectra for PdC-Me and PdC-H are found in the supplementary material (Figs. S7 and S8). Each depicted spectrum represents two current response peaks appearing at 2 and 5 Hz intermodulation frequencies which were analyzed to obtain the relevant corrosion kinetic parameters^64^. Electrochemical parameters such as corrosion current density $\left({i}_{corr}\right)$, Tafel constants (${\beta }_{a}$ and ${\beta }_{c}$), and the causality factors CF-2 and CF-3 were measured and listed in Table 9. It is found that the magnitude of ${i}_{corr}$ is suppressed by adding the investigated organic compounds in acidic solution and accordingly the inhibition efficiency is increased. This behavior indicates the effectiveness of the tested inhibitors due to the stability of the protective barrier layer on the steel surface^65^. The values of (${\beta }_{a}$ and ${\beta }_{c}$) were found to change with increasing concentration, therefore carbonitrile derivatives are mixed type inhibitors. The obtained values for the causality factors CF-2 and CF-3 were closed to a valid index confirming that the experimental results follow their hypothetical values (2 and 3)^66^.

**Table 9:** Electrochemical parameters obtained from EFM technique for the corrosion of carbon steel in 1 M HCl at various concentrations of carbonitrile compounds at 25 °C.

Comp.	Conc, M	i_corr._ μA cm^−2^	β_a_, mV dec^−1^	− β_c_, mV dec^−1^	CF(2)	CF(3)	%*η*
1.0 M HCl, Blank		299.2 ± 0.2009	93.7 ± 0.1453	101.3 ± 0.2027	1.89	2.96	–
PdC-OH	2 × 10^–5^	62.8 ± 0.2235	82.5 ± 0.1732	102.1 ± 0.2906	1.17	2.70	79.0
	4 × 10^–5^	56.4 ± 0.2127	83.6 ± 0.2309	111.6 ± 0.1732	1.65	2.63	81.2
	6 × 10^–5^	52.7 ± 0.2033	72.1 ± 0.1732	89. 7 ± 0.1763	1.97	3.22	82.4
	8 × 10^–5^	71. 5 ± 0.1764	74.0 ± 0.2309	87.0 ± 0.2082	1.40	2.71	88.6
	1 × 10^–4^	67.6 ± 0.1551	85.0 ± 0.2102	107.0 ± 0.2028	1.82	2.69	89.2
PdC-Me	2 × 10^–5^	91.8 ± 0.1614	93.8 ± 0.1732	104.3 ± 0.2028	1.97	3.20	69.3
	4 × 10^–5^	89.8 ± 0.1301	92.9 ± 0.2101	103.1 ± 0.2123	1.57	3.47	70.3
	6 × 10^–5^	68.5 ± 0.1564	79.0 ± 0.2423	93.1 ± 0.2234	2.22	2.91	77.1
	8 × 10^–5^	65.1 ± 0.2603	76.1 ± 0.2512	111.2 ± 0.2131	2.04	2.59	78.3
	1 × 10^–4^	64.20.2028	69.3 ± 0.1202	76.2 ± 0.2028	2.08	2.47	78.5
PdC-H	2 × 10^–5^	92.3 ± 0.2028	108.3 ± 0.2333	133.2 ± 0.1732	2.20	2.66	69.2
	4 × 10^–5^	88.9 ± 0.2906	95.4 ± 0.1453	110.7 ± 0.2309	1.90	2.70	70.3
	6 × 10^–5^	80.0 ± 0.1732	106.1 ± 0.2027	123.1 ± 0.1453	1.95	3.48	73.3
	8 × 10^–5^	70.6 ± 0.2028	96.7 ± 0.20223	129.8 ± 0.2028	1.88	2.99	76.4
	1 × 10^–4^	70.3 ± 0.2082	86.3 ± 0.1027	99.9 ± 0.1453	2.25	3.22	76.5

### Quantum chemical parameters

**Figure 11:**
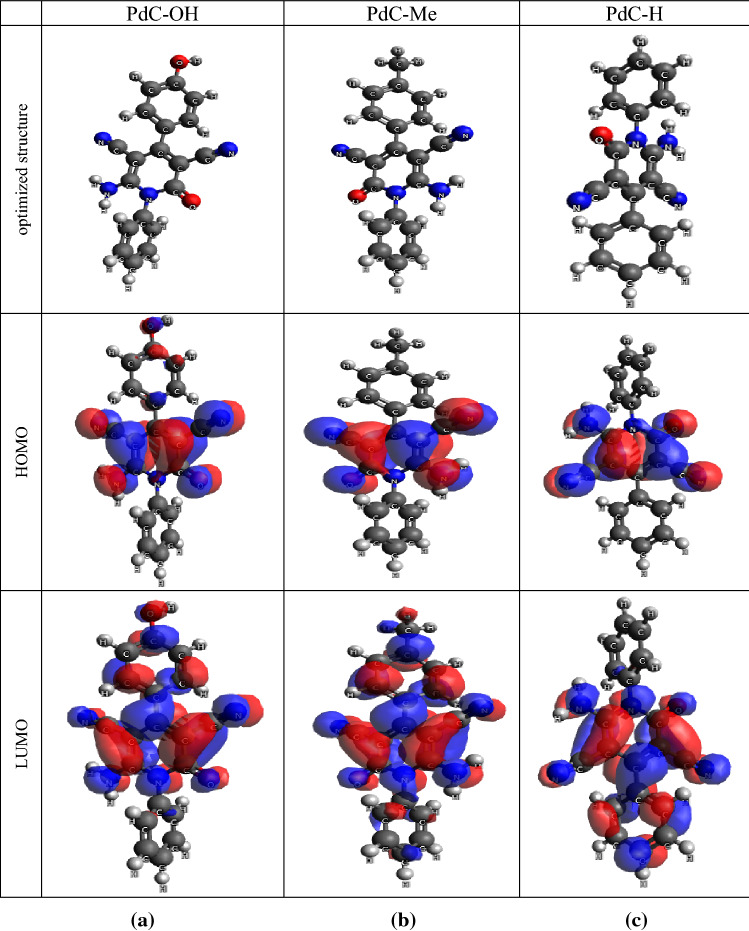
(**a**) Optimized molecular structure, (**b**) HOMO and (**c**) LUMO molecular orbital density distribution of PdC-OH, PdC-Me and PdC-H.

Quantum chemical calculations were performed to inspect the effect of the structural and the electronic properties of the material on the corrosion inhibition performance. Besides, to gain insights into the donor–acceptor interactions between inhibitor molecules and metal atoms. According to the frontier molecular orbital theory FMO, analysis of the density distributions of HOMO and LUMO can determine the donation-acceptance ability and molecular reactivity of the investigated inhibitors. E_HOMO_ denotes the ability of a molecule to donate electrons, whereas E_LUMO_ represents the ability of a molecule to accept electrons. Figure 11 shows the optimized molecular structures, HOMO and LUMO electronic density distributions, respectively. All the calculated quantum chemical parameters are listed in Table 10 including the energy values for the molecular orbitals (HOMO and LUMO), the energy gap (ΔE), and the dipole moment (μ). Examination of Fig. 11, the electron density of the HOMO orbitals of the carbonitrile derivatives is concentrated over the entire pyridine ring, and the high distribution on the phenolic ring in the PdC-OH compound. The contribution of the whole molecules in electron transfer is attributed to the presence of high electron density throughout the π-electrons of the aromatic and pyridine moieties, which are the sites most susceptible to electrophilic attacks in the molecules. However, it can also be seen that the presence of groups (–NH_2_–C= O and –CN) in the pyridine ring is a relatively softer part of the molecules, mainly involved in electron transfer. Moreover, the LUMO electron density of the three derivatives is mainly spread over the pyridine ring and the aromatic rings. Thus, the tendency to accept charges accumulated on the metal surface increases to form a feedback bond between the donor iron atoms and the acceptor anti-bonding orbital of the inhibitor^63,67^. Therefore, the determination of the electronic density of the HOMO and LUMO orbitals revealed that the studied inhibitors could adsorb on the steel surface by donating π-electrons from pyridine and aromatic moieties (Nucleophilic attack) to the vacant d-orbital of the metal and another possibility is that these derivatives may adsorb by acquiring electrons from the metal surface (Electrophilic attack)^68^. In Table 9, the calculated values for E_HOMO_ and E_LUMO_ show that PdC-OH has a relatively higher value for E_HOMO_ and a relatively lower value for E_LUMO_ compared to PdC-H and PdC-Me. This finding indicates that PdC-OH has the highest propensity to adsorb on the carbon steel surface. It is generally believed that molecules with low E_LUMO_ values and high E_HOMO_ values tend to present better inhibition efficiency^69^. The energy band gap ΔE (ΔE = E_HOMO_ − E_LUMO_)^70^ is the reactivity coefficient in theoretical studies. Murulana et al. postulated that the molecules which have a small energy gap value are considered highly reactive molecules and have good corrosion performance on the metal surface^71^. PdC-OH has the smallest value of ΔE as reported in Table 9. The dipole moment μ is a descriptor of the polarity in the covalent bond of molecules^71^. Abdallah et al.^72^ alluded that the dipole moment is an indication of the electronic distribution in the molecule. The efficiency of corrosion inhibition increases with increasing value of μ, due to stronger dipole–dipole interactions with the metal surface resulting in strong adsorption and effective corrosion inhibition^73^. The µ values follow the order: PdC-OH > PdC-Me > PdC-H which may be attributed to the largest value of the adsorption preference for the inhibitor molecule on the metal surface. The DFT results by DMol3 in Table 9 showed that the compound with the OH Phenolic group was the most reactive compound of the tested group. This observation is consistent with previous experimental methods. Classification of these inhibitors according to their inhibition efficiency is PdC-OH > PdC-Me > PdC-H.

**Table 10:** Quantum parameters of the investigated compounds at DFT level using Dmol3 and Orca.

Quantum parameter	PdC-OH	PdC-Me	PdC-H
**DFT calculations with DNP basis Set BOP/GGA by Dmol3**			
E_HOMO_, eV	− 5.160	− 5.172	− 5.185
E_LUMO_, eV	− 2.115	− 2.113	− 2.110
ΔE = (E_LUMO_ − E_HOMO_), eV	3.045	3.059	3.075
Dipole moment, Debye	12.156	11.825	11.627
**DFT calculations DEF2-SVP basis Set with B3LYP by Orca 4**			
E_HOMO_, eV	− 6.084	− 6.008	− 6.125
E_LUMO_, eV	− 1.786	− 1.872	− 1.831
ΔE = (E_LUMO_ − E_HOMO_), eV	4.298	4.136	4.294
Dipole moment, Debye	3.874	3.913	4.429

### Monte Carlo simulation

**Figure 12:**
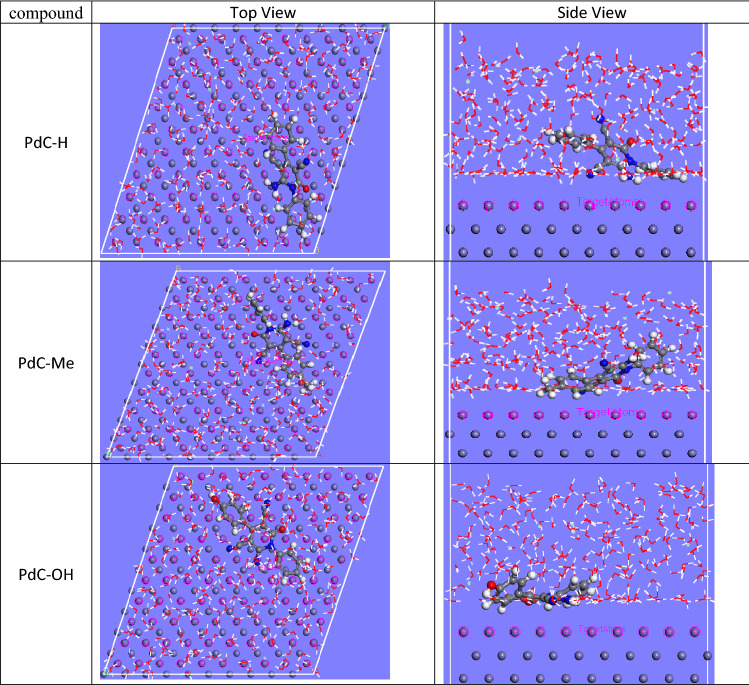
Top and Side Views for the most stable adsorption equilibrium position for studied compounds (PdC-H, PdC-Me and PdC-OH) on the carbon steel.

Monte Carlo simulations are performed to describe how inhibitor molecules behave and interact at a metal interface. This method can effectively help reduce the cost of experiment, and can also help in the experimental design of inhibitor molecules that effectively inhibit metal corrosion^74^. Figure 12 shows a side view and a top view of the optimized equilibrium configurations of the three carbonitrile derivatives adsorbed on a carbon steel substrate. Considering this output in Fig. 12, the geometrically optimized molecules under study being loaded on Fe surfaces indicated that these molecules tended to adsorb almost planar orientation on the surface. This flat orientation provides close contact with the active sites to impede the corrosion reaction^75^. The results in Table 11 give the output of energies computed by MC simulation such as total adsorption, adsorption energy, rigid absorption, and deformation energies. The outlined adsorption energy (E_ads_) was calculated mathematically by the summation of the rigid adsorption energy and the deformation energy of the adsorbate molecules^76,77^. In general, E_ads_ is used to express the strength of the adsorption process of inhibitors on the surface of iron. The rigid adsorption energy was described as the energy released when the inhibitor molecules are adsorbed on the metal surface and the deformation energy recognized as the energy released when the adsorbed-adsorbate components undergo relaxation on the surface^65^. As depicted in Table 11 the order of the calculated E_ads_ values is PdC-OH (− 4073.86) > PdC- Me (− 4024.09) > PdC-H (− 4006.85), indicating the inhibition performance follows: PdC-OH > PdC-Me > PdC-H. Negative magnitudes of E_ads_ for all three studied inhibitors denote the spontaneous and strong adsorption process that occurred on the iron substrate. As shown in Table 11, PdC-OH reached a maximum value of dE_ads_/dNi (− 208.85) in the simulation method indicating its highest contribution to the total adsorption energy^78^. This theoretical study supports previous experiments and proves coincident well with all acquired data. It can be concluded that all the three studied inhibitors can adsorb on the steel surface through the π-charge of pyridine and the moieties of the aromatic ring providing strong bonding to the metal surface. Finally, the use of carbonitrile derivatives as a corrosion inhibitor has a great potential for corrosion prevention**,** and consequently better inhibition efficiency.

**Table 11:** List of Monte Carlo simulations parameters of compounds on Fe.

Structures	PdC-H/Fe	PdC-Me/Fe	PdC-OH/Fe
Total energy	− 4073.22	− 4098.56	− 4155.66
Adsorption energy	− 4006.85	− 4024.09	− 4073.86
Rigid adsorption energy	− 4186.77	− 4196.86	− 4253.11
Deformation energy	179.93	172.77	179.25
Inh: dEad/dNi	− 142.31	− 142.00	− 208.85
H_2_O: dEad/dNi	− 7.60	− 12.35	− 13.45
H_3_O^+^: dEad/dNi	− 151.50	− 141.68	− 155.51
Cl^−^: dEad/dNi	− 153.12	− 131.71	− 143.51

### Atomic force microscopy (AFM) analysis

**Figure 13:**
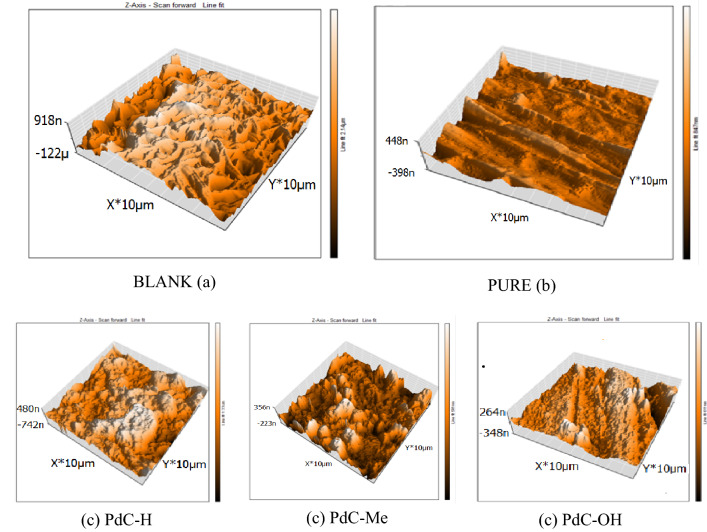
AFM images for uninhibited (**a**), pure (**b**) and inhibited carbon steel (**c**) after immersion for 24 h in 1 M HCl with (PdC-H, PdC-Me and PdC-OH) compounds.

In the field of corrosion research, AFM analysis has been used to elucidate the effect of inhibitors on corrosion product development or corrosion progression at the metal/solution interface. The resulting topographical images directly reflect the surface of carbon steel at the nanometer scale^79^. AFM characterizes the morphology of the corroded metal in 3D images. Figure 13a shows a micrograph from AFM analysis of a carbon steel surface after immersion in 1 M HCl for 24 h in the absence of an optimal concentration of inhibitors^72^. The surface was severely damaged and corroded by acid attack, which was estimated by the average roughness (Ra) that recorded a height of 272.8 nm. Figure 13b shows the smooth and uniform surface of the free sample with Ra 49.8 nm^72^. However, the average roughness value was reduced to 152.11, 76.06, 64.55 nm after the treatment of the test solution with carbonitrile derivatives PdC-H, PdC-Me, and PdC-OH, respectively as shown in Fig. 13c compared to the absence of these inhibitors. The improvement shown in the surface topography is due to the adsorption of the investigated carbonitrile molecules onto the steel surface and the formation of a protective layer. In view of the above findings, the inhibition action tendency of inhibitors acquired from surface analysis is related to those acquired from experimental results.

### Attenuated total refraction infrared (ATR-IR) analysis

**Figure 14:**
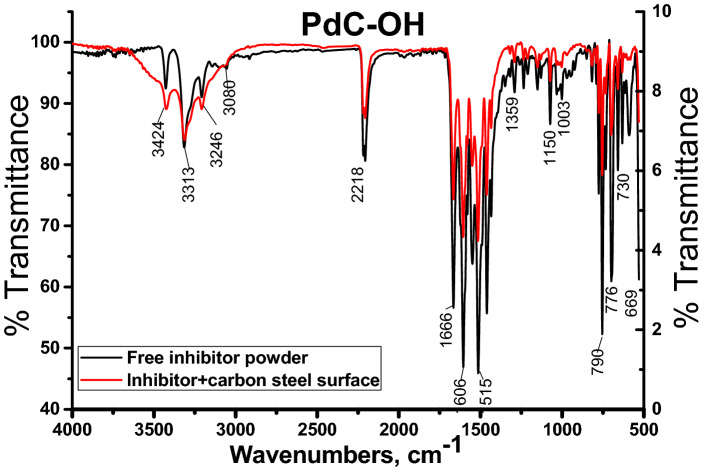
ATR-IR spectra of pure PdC-OH compound and carbon steel metal surface in 1 M HCl + PdC-OH.

This method concerns identifying the adsorbed functional groups of the organic compounds upon the metal substrate, ATR-IR was performed with a range of 4000 to 400 cm^−1^. Figure 14 signifies the ATR-IR spectrum of the PdC-OH compound and the construction of a protective film on the carbon steel surface after soaking for 24 h in 1 M HCl with the optimum concentration 10^–4^ M of this compound. ATR-IR spectra for PdC-Me and PdC-H are shown in are found in the supplementary material (Figs. S9 and S10). It can be seen in Fig. 13 that the ATR-IR spectra of the protective film formed on the steel surface showed all the characteristic peaks of the pure inhibitor, indicating that the inhibitor adsorbed on the steel surface. The characteristic peaks of the active function groups of the free organic compound before (pure inhibitors) and the other peaks in the presence of this compound are discussed and summarized in Table 12. From the obvious peaks, function groups such as (Nitrile C≡N, –C = O and –NH–) appear on the carbon steel surface. Besides, there were small changes, and some frequencies of weak function groups were shifted significantly such as the peak of (C–O) Phenolic and stretching (C=C) aromatic as shown in Table 12. The above findings clearly illustrate that the specific hetero-aromatic ring (–OH) and the π electrons of the aromatic rings were involved in the adsorption process of the PdC-OH compound.

**Table 12:** Characteristic peaks before and after immersion in1M HCl in presence of the PdC-OH derivative for 24 h on the carbon steel surface at 25 °C.

Function group	Characteristic absorption (s) cm^−1^	
	(PdC-OH)	
	Before	After
Stretching (NH–)	(3427, 3314)	(3324, 3314)
Stretching (NH–)	3055	3050
Stretching (C–H) sp^2^	–	–
Stretching (C–H) sp^3^	3246	3246
stretching –OH	2218	2218
Nitrile (C≡N)	1666	1666
Stretching (–C=O)	1606	1606
Bending (–NH–)	1515	1514
Stretching (–C=C) aromatic	1359	1349
(C–O) Phenolic	(868, 749, 666)	(838, 750, 594)
Aromatic bending (C–H) sp^2^	(1150, 1003)	(1151, 1002)
Aromatic substituted	(790, 776, 730, 669)	(792, 755, 733, 659)

### X-ray photoelectron spectroscopy analysis

**Figure 15:**
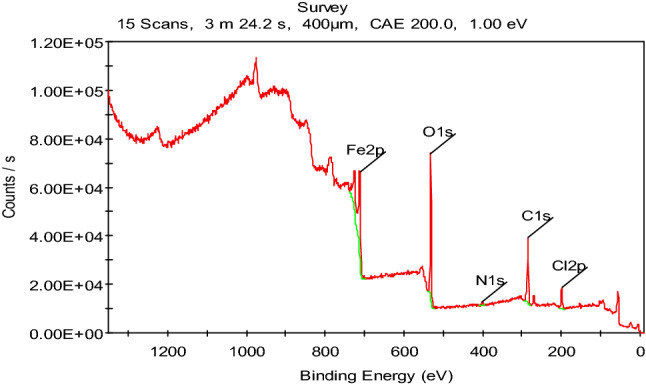
The XPS survey spectrum of PdC-OH compound adsorbed on the carbon steel in 1 M HCl at 25 °C.

**Figure 16:**
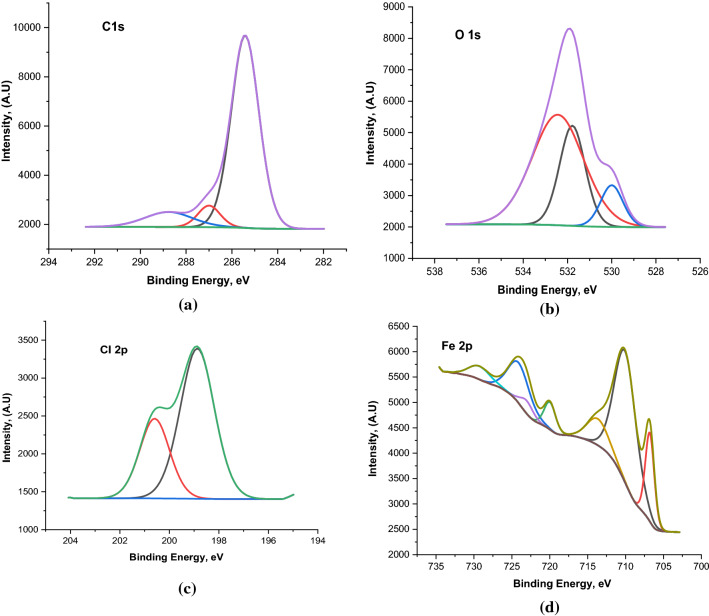
High-resolution X-ray photoelectron deconvoluted profiles of (**a**) C 1 s, (**b**) O 1 s, (**c**) Cl 2p, and (**d**) Fe 2p for carbon steel in 1 M HCl.

**Figure 17:**
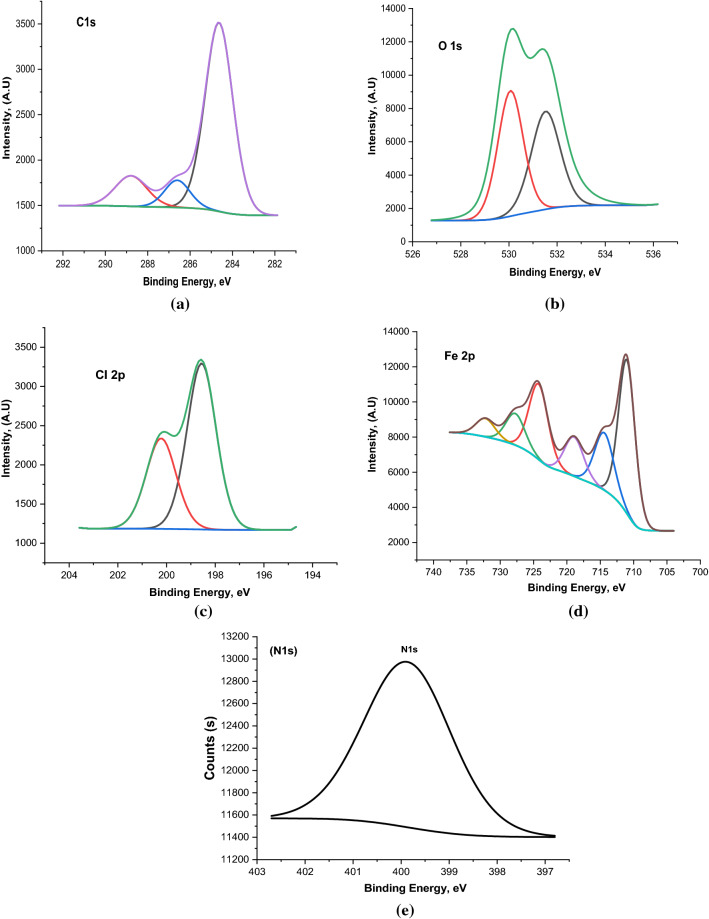
High-resolution X-ray photoelectron deconvoluted profiles of (**a**) C 1 s, (**b**) O 1 s, (**c**) Cl 2p, (**d**) Fe 2p, and (**e**) N 1s for carbon steel in 1 M HCl + carbonitrile compounds.

X-ray photoelectron spectroscopy (XPS) was performed to confirm the adsorption of the studied organic compounds on the carbon steel surface and to determine the chemical nature of the inhibitors/carbon steel interface. Figure 15 shows the high-resolution XPS spectrum survey obtained for the surface of corroded carbon steel in 1 M HCl solution in the presence of the PdC-OH derivative. The XPS spectrum shows complex forms, which were assigned to the corresponding species through a deconvolution fitting procedure. High-resolution XPS spectra obtained for carbon steel surface corroded in 1 M HCl composed of (Fe 2p, O 1s, Cl 2p, C 1s) are illustrated in Fig. 16. While in the presence of the studied carbonitrile compounds, the XPS spectra consisted of the same elements (Fe 2p, O 1s, Cl 2p, C 1s) in addition to N 1s core level as shown in Fig. 17. The XPS spectrum of Fe 2p shows six peaks, the higher peak at low binding energy (711.2 eV) corresponding to metallic iron^80^. The peak at 714.6 eV is attributed to Fe 2p3/2, and the small peak at 719.40 eV is ascribed to the Fe^3+^satellite^81^. In addition, the peaks at 724.3 eV ,and 727.9 eV can be attributed to Fe 2p1/2 due to the presence of iron in the form of Fe_3_O_4_, α-Fe_2_O_3_ and FeOOH and also the involvement of Fe^3+^ in the complex formation with the inhibitor molecules^82^. The last peak at 732.4 eV is related to the oxidation of the steel surface. The C 1s spectra of the carbon steel in HCl alone and with the studied compounds show two characteristic peaks at the binding energy 284.6 eV and 286.2 eV assigned to a C–C bond and C=O bond, respectively while in the case of only the investigated carbonitrile derivatives there are more peaks observed at 288.4 eV which is attributed to the sp^2^-hybridized carbon^83,84^ which comes from the inhibitors molecules. XPS spectra of O 1s in blank solution show three peaks one of which is at BE 530.0 eV which is attributed to iron oxide (FeO and Fe_2_O_3_^85,86^. The second has a binding energy of 531.8 eV, related to hydroxide bonds chemisorbed on the surface^87,88^. The third peak, at 532.4 eV, can be assigned to the oxygen of the adsorbed water and OH^−^ in FeOOH^89^. In the case of the inhibited samples, the oxygen spectrum shows three peaks at binding energies of 530.8 assigned to iron oxide, the peak at 532.8 for OH^−^ in FeOOH, and the last peak at 534.2 corresponding to C–OH and surface adsorbed-water molecules. Moreover, in the presence of the investigated inhibitors, the O1s core level signal decreases significantly which is consistent with the adsorption of the inhibitors on the steel surface. Also, the XPS spectrum for Cl 2p shows the best fit in two components locate at around 198.9 eV for Cl 2p3/2 and 200.6 eV for Cl 2p1/2 as reported by Gu et al.^90^.The same peaks are obtained in the absence and presence of the studied inhibitors due to the arrival of some chloride ions to the surface and are responsible for the corrosion of the alloy. Finally, the XPS spectrum of N 1s appears with a single peak at 399.9 eV, and this peak can be attributed to the neutral imine (–N=) and amine (–N–H) nitrogen atoms as previously reported^91^. The appearance of N peak in the spectra of the protected sample surface confirms the adsorption of the studied inhibitors on the sample surface. According to the XPS results, we can conclude that the composite film formed on the surface contains iron oxide/hydroxide and carbonitrile compounds. These components provide a protective film that can effectively isolate the corrosion medium and reduce the corrosion of carbon steel.

### Possible corrosion inhibition mechanism

The adsorption process of organic inhibitor molecules depends on many physical and chemical properties such as; Electron density, chemical structure, metal nature, charges at the metal/solution interface, and type of aggressive medium (pH and/or electrode potential)^92,93^. These properties affect the mode the molecules interact on the metal surface. Adsorption of organic molecules on solid surfaces cannot be considered purely physical or chemical; A combination of both processes can occur in adsorption^83,84^. Physical interactions are accepted as the first step for adsorption of molecules on the metal surface and then chemical adsorption may occur via different charge sharing processes^94^. In a solution of hydrochloric acid, the inhibitor molecules are adsorbed on the metal surface through the following interactions: (i) the electrostatic interaction between the positively protonated inhibitor and the chloride ions adsorbed on the metal surface (physisorption process); (ii) the chemical interaction between the lone-pair electrons on the heteroatoms (N, S, O, P) and the unoccupied d-orbital on the metal surface (chemical adsorption process); (iii) the donor–acceptor interaction between π-electrons of the aromatic ring and the vacant d-orbitals on the metal surface (chemical adsorption process); and (iv) Retro-donation interaction between the excess negatively charged metal surface and the π*anti-bonding of the inhibitor molecule. To elucidate the corrosion inhibition mechanism of the investigated carbonitrile derivatives, the inhibition action is due to adsorption of these compounds at the metal/solution interface. Adsorption may occur through a donor–acceptor interaction between conjugated π-charge of (two aromatic and pyridine rings moieties) and an unoccupied d-orbital of iron atoms to form coordination bonds (chemical adsorption process)^95^. The electron density of the donor atom in the carbonitrile functional group depends on the substituents present in these compounds. The direction of the inhibition potentials of three derivatives is determined by the value of Hammett sigma constant (σ) for the substituent groups (OH, CH_3_ and H). This is because; in this type of derivatives the adsorption center is conjugated with the ring. The presence of electron donation (OH, CH_3_) (σ = − 0.17 for p-CH_3_ and σ = − 0.37 for p-OH) increases the electron density of the neighboring aromatic ring and makes the π-electrons more available for interaction with the C-steel surface thus strengthens the adsorption of PdC-OH and PdC-Me on the steel surface. PdC-OH has the highest inhibition efficiency, which is due to the presence of the OH group which added an extra adsorption center to the molecule compared to the CH_3_ group in PdC-Me compound. PdC-H ranked below the two inhibitors in the inhibition order due to the presence of a hydrogen atom (H-atom with σ = 0.0) which is considered an electron-withdrawing atom^87^. Meanwhile, the carbonitrile derivatives can accept electrons from the d-orbital of iron atoms through their π* anti-bonding orbital to form a feedback bond (retro-donation process), thus promoting the adsorption of the inhibitor molecules on the steel surface. In addition, the carbonitrile molecules may be adsorbed on the metal surface through the van der Waals force by interacting neutral inhibitor molecules with iron ions to form [Fe-PdC] complexes (physical adsorption process).

14 $$Inh+Fe\left(0\right)\to \left[Fe\left(0\right)Inh\right]$$

It can be concluded that the good inhibition efficiency of the studied derivatives is due to the presence of two aromatic and pyridine rings, the polar functional groups such as (–CN, –NH_2_) that act as adsorption centers, and the specific hetero-aromatic ring (–OH) that present in PdC-OH compound. Hence, the type of adsorption of carbonitrile derivatives on the C-steel surface is more than just physical adsorption but not purely chemical^88–90^.

## Conclusion

According to the present study, the synthesized carbonitrile compounds can be used as effective corrosion inhibitors for C-steel in 1 M HCl. The corrosion inhibition efficiency increases with increasing concentrations of the studied inhibitors, and ranked as PdC-OH > PdC-Me > PdC-H. The adsorption of the inhibitors on the surface of C-steel in 1 M HCl follows the Langmuir isotherm and the calculated thermodynamic parameters propose that the adsorption is predominantly chemisorption. Addition of KI to the PdC-OH shows a synergistic effect that significantly improves its inhibition efficiency. The magnitudes of the synergism parameter (Sө) showed that the corrosion inhibition produced by PdC-OH and iodide mixture is synergistic in nature. Tafel polarization data showed that the corrosion current density decreases and the corrosion potential changes slightly with the addition of carbonitrile compounds, therefore these compounds are mixed type of inhibitors. The surface examined by ATR-IR, AFM, and XPS showed the formation of adsorbed film on C-steel surface. There is a good correlation between theoretical and experimental data.

## Supplementary Information

Supplementary Information.
