# Supplementary material for: Experimental and computational chemical studies on the corrosion inhibitive properties of carbonitrile compounds for carbon steel in aqueous solutions
Source: Sci Rep. 2021 Nov 4;11:21672. doi: 10.1038/s41598-021-00701-z (PMC8569179; doi:10.1038/s41598-021-00701-z)
Supplement: Supplementary file 1 — Supplementary Information. [file 41598_2021_701_MOESM1_ESM.doc]

**Experimental and computational chemical studies on the corrosion inhibitive properties of Carbonitrile compounds for carbon steel in aqueous solutions**

Abdelaziz S. Fouda^1^, Abdelmonem H. El-Askalany^1^, Ahmed F.S. Molouk^1^, Niveen S. Elsheikh^1^, Ashraf S. Abousalem* ^1,2^

^1^ Chemistry Department, Faculty of Science, Mansoura University, El-Mansoura, Egypt

^2^Quality Control Laboratory, Operations Department, JOTUN, EGYPT. Email: ashraf.abouslaem@gmail.com

Fig. S1. Weight loss-time curves for the dissolution of carbon steel in 1 M HCl without and with various concentrations of PdC-Me and PdC-H at 25^o^C.

Fig. S2. Difference of %η against different solution temperatures for compounds PdC-Me and PdC-H.

Fig. S3. Arrhenius plots (log k vs 1000/T) for carbon steel in 1M HCl without and with various concentrations of PdC-Me and PdC-H compounds.

Fig. S4. Potentiodynamic polarization curves for the corrosion of carbon steel in 1M HCl without and with different concentrations of PdC-Me and PdC-H compounds at 25°C.

| (a) | (b) |
| --- | --- |

Fig. S5. Nyquist (a) and Bode-phase angle (b) plots for corrosion of carbon steel in 1M HCl in the absence and presence of various concentrations of PdC-Me compound at 25^O^C.

| (a) | (b) |
| --- | --- |

Fig. S6. Nyquist (a) and Bode-phase angle (b) plots for corrosion of carbon steel in 1M HCl in the absence and presence of various concentrations of PdC-H compound at 25^O^C.

Fig. S7. EFM spectra of carbon steel in 1M HCl without and with various concentrations of PdC-Me compound at 25°C.

Fig. S8. EFM spectra of carbon steel in 1M HCl without and with various concentrations of PdC-H compound at 25°C.

Fig. S9. ATR-IR spectra of pure PdC-Me compound and carbon steel metal surface in 1M HCl + PdC-Me.

Fig. S10. ATR-IR spectra of pure PdC-H compound and carbon steel metal surface in 1M HCl + PdC-H.
